# Patient and Family Perspectives on Clinical Outcomes in Therapy Trials in Megalencephalic Leukoencephalopathy with Subcortical Cysts: An Open Inventory

**DOI:** 10.1055/a-2851-9956

**Published:** 2026-04-23

**Authors:** N. R. A. Versaevel, A. Chapleau, P. Topaloğlu, F. Nicita, J. Sinha, G. Bernard, A. Fatemi, P. Sgobbi, E. M. C. Hamilton, C. Marouda, R. van Eekelen, M. S. van der Knaap

**Affiliations:** 1 Department of Child Neurology, Amsterdam Leukodystrophy Center, Amsterdam University Medical Center, Emma Children's Hospital, Amsterdam Neuroscience, Amsterdam, The Netherlands; 2 Child Health and Human Development Program, Research Institute of the McGill University Health Centre, Montreal, Quebec, Canada; 3 Department of Neurology and Neurosurgery, McGill University, Montreal, Quebec, Canada; 4 Alliance MLC; 5 Division of Child Neurology, Department of Neurology, Istanbul Faculty of Medicine, Istanbul University, Istanbul, Türkiye; 6 Unit of Muscular and Neurodegenerative Diseases, Bambino Gesù Children's Hospital, IRCCS, Rome, Italy; 7 Department of Pediatric Neurology, Center for Neurosciences and Research, NH Hospital, RN Tagore International Institute, Kolkata, India; 8 Division of Medical Genetics, Department of Neurology and Neurosurgery, Pediatrics, and Human Genetics, Department of Specialized Medicine, McGill University Health Centre, Child Health and Human Development Program, Research Institute of the McGill University Health Center, McGill University, Montreal, Quebec, Canada; 9 Department of Neurology and Developmental Medicine, Kennedy Krieger Institute, Baltimore, Maryland, United States; 10 Department of Neurology, PSEG Centro de Pesquisa Clínica, São Paulo, São Paulo, Brazil; 11 Department of Epidemiology and Data Science, Amsterdam University Medical Center, Amsterdam, The Netherlands; 12 Department of Integrative Neurophysiology, Center for Neurogenomics and Cognitive Research, Vrije Universiteit Amsterdam, Amsterdam Neuroscience, Amsterdam, The Netherlands

**Keywords:** megalencephalic leukoencephalopathy with subcortical cysts, trial readiness, patient-centered outcomes, caregiver perspectives, patient perspectives

## Abstract

**Objective:**

To determine which clinical outcomes are most meaningful to patients with megalencephalic leukoencephalopathy with subcortical cysts (MLC) and their caregivers to enhance clinical trial readiness.

**Methods:**

An online survey was developed by the MLC Clinical Expert Consortium and Alliance MLC. The survey, available in eight languages, was distributed via treating neurologists and Alliance MLC, and completed anonymously by patients and caregivers. It included open-ended questions on burdensome daily-life aspects, treatment priorities, and therapy expectations; responses were categorized into functional domains. The survey also included a ranking of key symptom domains. Responses were analyzed separately for caregiver- and patient-reported burden. Subgroup analyses were performed for age (<15 years vs. ≥15 years) and country of residence (Türkiye, India, and Italy).

**Results:**

Eighty-five surveys representing 83 patients were included. Motor dysfunction was most frequently reported as most burdensome for both caregivers (74%) and patients (58%), followed by communication and activities of daily living (ADL). Within the motor domain, ambulation was most frequently mentioned. Regarding the ranking, motor function scored highest in 69% of respondents. In patients ≥ 15 years, ambulation was less frequently mentioned, whereas ADL dependency was more frequently reported than in patients <15 years. Comparisons between countries revealed few differences. Hopes for therapy were mainly concerned with motor improvement and halting disease progression.

**Conclusion:**

Motor function, particularly ambulation, is the most burdensome and prioritized domain for patients with MLC and their caregivers, regardless of age or country. Incorporating patient-centered priorities into future clinical trial design is essential to ensure that emerging therapies target meaningful improvements.

## Introduction

Megalencephalic Leukoencephalopathy with subcortical Cysts (MLC) is an ultra-rare genetic brain disorder with infantile onset, defined by MRI evidence of chronic brain white matter edema and subcortical cysts in the anterior temporal region. 1 2 MLC occurs worldwide, with a relatively high prevalence in India and the Middle East. 3 4 5 MLC is caused by pathogenic variants in MLC1, GLIALCAM, GPRC5B, and AQP4. 6 7 8 9 Two different clinical and MRI phenotypes can be distinguished: classic and remitting. Classic MLC comprises approximately 85% of all patients 9 and is characterized by permanent white matter abnormalities on MRI and neurological decline. Clinical characteristics include macrocephaly with onset before 6 months of age, normal or mildly delayed early development, epilepsy, delayed onset progressive motor decline due to ataxia and spasticity, eventually leading to wheelchair dependency, and cognitive decline. 1 3 10 The remitting phenotype comprises approximately 15% of patients. 9 In this subtype, MRI abnormalities improve or resolve; there is no motor or cognitive decline, but intellectual disability and autism often persist. 10 11

At present, no curative treatment is available for MLC, and management remains symptomatic. Recently, however, advancements in gene therapy and drug-based approaches have shown potential for curative treatments in MLC. 8 12 These developments highlight the need for appropriate clinical outcome measures to evaluate therapeutic effectiveness. In this respect, it is important to consider input from patients and patients' family members, as they offer valuable perspectives on daily functioning, quality of life, and meaningful treatment outcomes. 13 In the current study, we present data obtained from a survey completed anonymously by MLC patients and their caregivers from all over the world. Our aim is to identify which clinical outcomes matter most to patients and their families, with the goal of guiding future therapy evaluation toward outcomes that align with their needs and priorities. By using mostly open-ended questions, we aim to create an inventory that captures the perspectives of patients with MLC and their families.

## Methods

### Survey and Respondents

The survey, available in Supplementary File (available in the online version only), was created by the international MLC clinical expert consortium, consisting of seven pediatric or adult neurologists, in collaboration with patient advocates from Alliance MLC (alliamcemlc.org). The survey was built in SurveyMonkey. 14 It was available in English, Turkish, French, Spanish, Portuguese, Italian, Dutch, and German; translations were made by the consortium members or colleague neurologists. Families received the link to the Survey from their neurologists or via the website of Alliance MLC. The survey was open to patients and families from March 5, 2025, to June 30, 2025. Ethics approval for the study was obtained from the institutional review board of the Amsterdam University Medical Center, where the study was initiated.

The surveys were completed anonymously by parents, legal guardians, and intellectually competent MLC patients. No age or ethnicity restrictions were applicable. The survey included four open-ended questions that allowed families and patients to respond freely. The respondents were asked to describe the most burdensome aspects of daily life, whether these issues should be considered when evaluating treatment outcomes, and to share their hopes and expectations for therapy. The question about the most burdensome aspects of daily life was divided into two parts: one for the caregivers' perspective and one for the patients' perspective, as perceived by the caregiver or the patient, depending on who filled in the survey. In cases where the patient was the respondent, the caregiver's part was left open. Additionally, respondents were able to comment on any items they felt were missing from the survey. Respondents also ranked predefined symptom domains by perceived importance. Within each domain, they could specify which particular symptoms they considered most important.

### Analysis

Only fully completed surveys were included in the analysis. Responses were translated into English and analyzed. Based on the responses, functional domains were defined, and responses were categorized into these domains. Separate analyses were performed for domains rated as burdensome to caregivers and for domains rated as burdensome to patients.

Patients < 15 years of age are generally less severely disabled than patients ≥ 15 years, and the degree of disability may influence the responses. Therefore, binary logistic regression analysis was performed for two patient age groups: <15 and ≥15 years. Binary logistic regression analysis was also performed for the three largest cohorts: Turkish, Indian, and Italian, as different cultural subgroups may respond differently. Türkiye, representing the largest patient group, was used as a reference. Odds ratios (ORs) and 95% confidence intervals (95% CI) were calculated for Italy versus Türkiye and India versus Türkiye. A *p* -value < 0.05 was considered statistically significant. When cell counts were < 5 or equal to zero, no reliable OR and CI could be calculated. These instances are indicated as “–” in the tables.

## Results

### Surveys

**Fig. 1:**
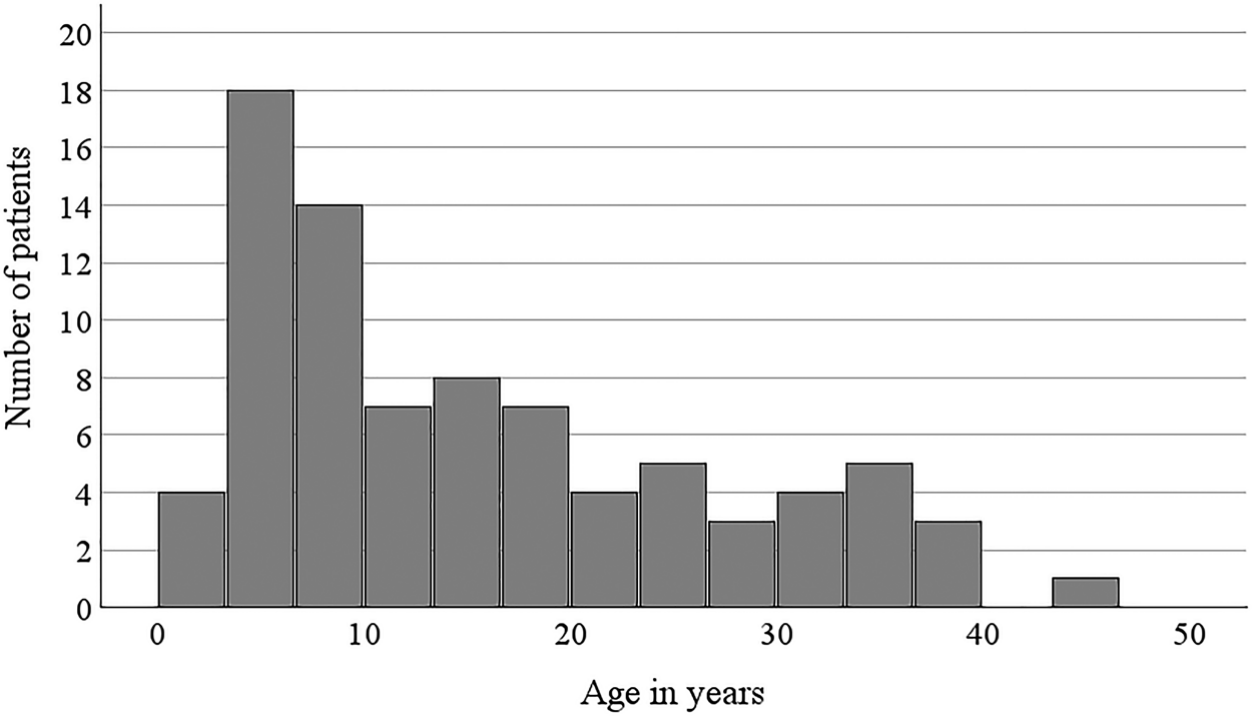
Age distribution of MLC patients included in the survey. The total number of MLC patients is 83. The median age is 13 years, with a first quartile (Q1) of 6 years and a third quartile (Q3) of 24 years.

A total of 117 individuals started the survey, but for 32 surveys, respondents only entered their own characteristics and did not respond to the survey questions; these were therefore excluded. The remaining 85 surveys were complete and included in the analysis. Two patients had surveys completed by both parents, bringing the total number of unique patients included in the study to 83. The respondents were mothers ( *n* = 49), fathers ( *n* = 27), legal guardians ( *n* = 4), and patients ( *n* = 5). The countries of patients' residence are listed in Supplementary Table S1 (available in the online version only). The majority of the respondents were based in Türkiye ( *n* = 23), Italy ( *n* = 17), and India ( *n* = 16). The age for MLC patients was skewed toward lower ages, as shown in Fig. 1 . The median age was 13 years (interquartile range: 6–24) in the overall population, 9 years (6–27) for the Turkish population, 19 years (15–34) for the Italian population, and 12 years (5–17) for the Indian population.

### Open Question on Most Burdensome Symptoms

**Fig. 2:**
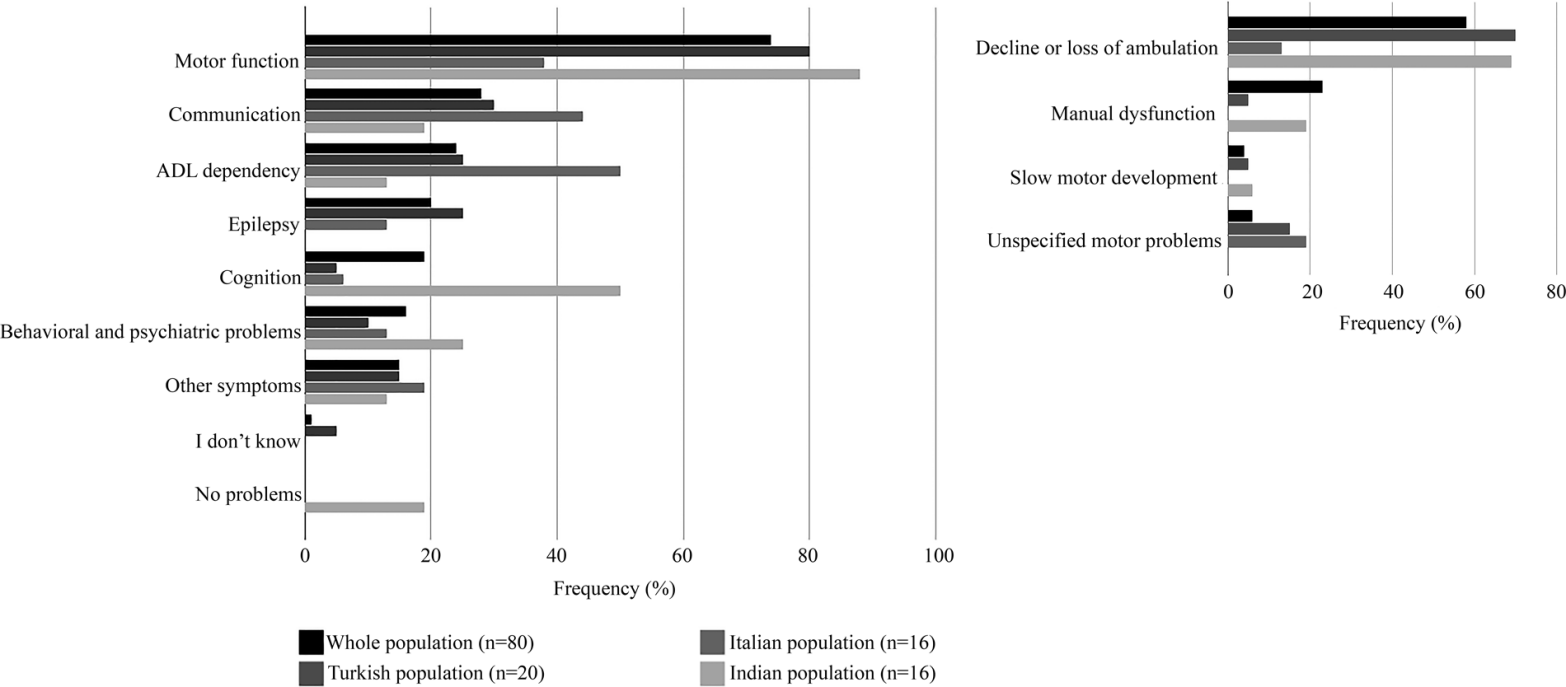
Most burdensome symptoms related to MLC in daily life for caregivers in different populations. The frequency of reports per domain is shown on the *x* -axis, and domains are displayed on the *y* -axis. Domains comprising motor function are specified separately. This figure is supported by Supplementary Table S2 (available in the online version only).

**Fig. 3:**
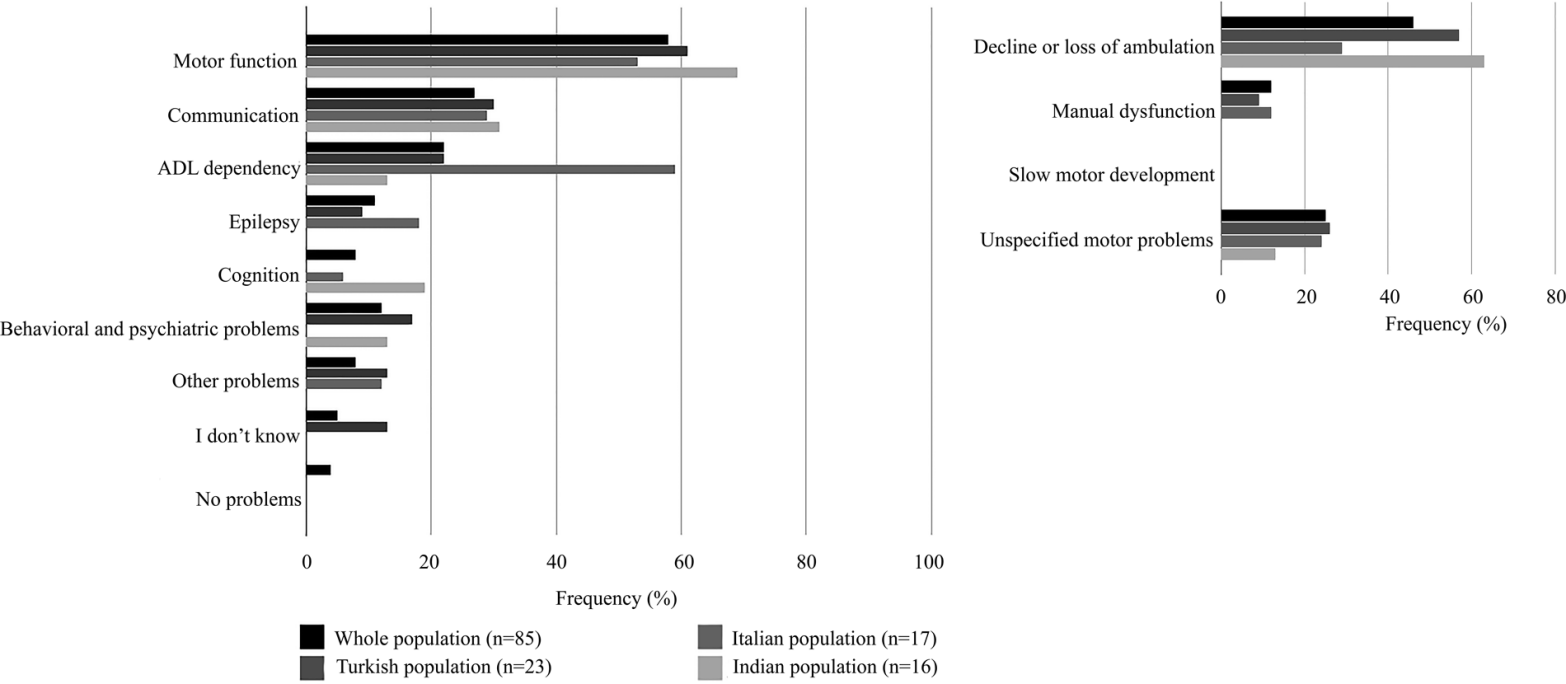
Most burdensome symptoms related to MLC in daily life for patients in different populations. The frequency of reports per domain is shown on the *x* -axis, and domains are displayed on the *y* -axis. Domains comprising motor function are specified separately. This figure is supported by Supplementary Table S3 (available in the online version only).

Figs. 2 and 3 and Supplementary Tables S2 to S4 (available in the online version only) summarize the MLC-related symptom domains caregivers considered most burdensome in their own daily lives and in the daily lives of patients. Responses from the five patients were included in the latter for analysis; their individual responses are also specified below. Respondents could report multiple symptoms. The numbers and percentages reflect how frequently each symptom was mentioned. When asked to identify the most burdensome symptom, caregivers most frequently mentioned motor dysfunction, both in relation to their own daily lives (74%) and the daily lives of patients (58%). Three of the five MLC patients who answered the questionnaire were included within the latter group of 58%. Within the motor domain, four subdomains were defined to reflect the variety of responses given by participants: decline or loss of ambulation, manual dysfunction, unspecified motor problems, and slow motor development. The subdomain of decline or loss of ambulation included references to walking difficulties, mobility limitations, wheelchair dependency, impaired balance, leg dysfunction, and gross motor impairment. Unspecified motor problems included concerns with unspecified functional impact on motor performance, such as ataxia, spasticity, muscle spasms, muscle loss, and general statements about motor dysfunction. Slow motor development refers to the delayed acquisition of developmental motor milestones. Approximately half of the respondents specifically mentioned ambulation ( Supplementary Tables S2 and S3 , available in the online version only). Communication was the second most frequently mentioned domain (28% for the life of caregivers and 27% for the life of patients) and encompassed statements about verbal and nonverbal communication and speech and language difficulties. Activities of daily living (ADL) were the third most mentioned domain (24% for caregivers and 22% for patients) and contained statements regarding eating and drinking, washing, toileting, dressing, and complete ADL dependency. Epilepsy was fourth (20 and 11%), with statements mostly concerning seizure frequency. Cognitive functioning was the fifth (19 and 8%) and included references to learning difficulties, memory, comprehension, awareness of situations, recognition of needs and emotions, rational decision-making, processing speed, attention, concentration, and cognitive regression. Behavioral and psychiatric problems were the sixth (16 and 12%) and were described in terms of autism or autistic like features, bipolar disorder, crying fits, aggression, anger, agitation, social functioning, tics, pronounced positive or negative character traits, and coprophagia. All other reported characteristics, comprising head trauma, fears and worries, sleep problems, pain, fatigue, and urinary or bowel issues, were classified under “other symptoms.”

### Ranking

An overview of the ranking of the domains by the respondents is given in Table 1 . Motor function was ranked as the highest priority by 69% of the respondents, followed by cognition (15%) and epilepsy control (8%). Pain was most frequently rated as least important, with 45% of the respondents, followed by sleep (18%) and bladder and bowel control (14%). Supplementary Table S6 (available in the online version only) presents the responses of patients ( *n* = 5) and caregivers ( *n* = 80) regarding ranking separately. Patients ranked motor function as most important and ranked control of epilepsy as least important.

**Table 1:** Ranking of the most and least important domains in the whole population and different age groups

		Whole population ( *n* = 85)	Population < 15 y ( *n* = 49)	Population ≥ 15 y ( *n* = 36)
Rank	Domain	Number (%)	Number (%)	Number (%)
1	Motor function	59 (69)	35 (71)	24 (67)
2	Cognitive function	13 (15)	8 (16)	5 (14)
3	Control of epilepsy	7 (8)	4 (8)	3 (8)
…				
7	Bladder and bowel control	12 (14)	9 (18)	–
8	Sleep	15 (18)	9 (18)	6 (17)
9	Pain	38 (45)	21 (43)	17 (47)

Note: Data are presented as numbers (%). “…” indicates the ranks in between. Domains include motor function, cognitive function, epilepsy control, sleep, pain, and bladder/bowel control. The domains not shown in the table are mood, behavior, and communication.

### Effect of Age

**Fig. 4:**
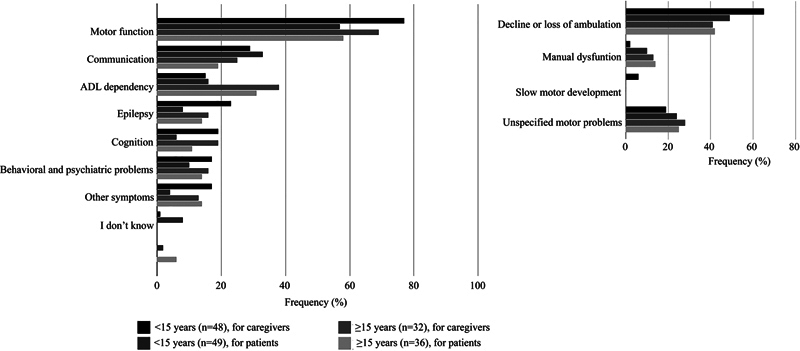
Most burdensome symptoms related to MLC in daily life in different age groups. The frequency of reports per domain is shown on the *x* -axis, and domains are displayed on the *y* -axis. Domains comprising motor function are specified separately. This figure is supported by Supplementary Table S4 (available in the online version only).

When comparing caregiver responses for age groups < 15 years versus ≥15 years ( Fig. 4 ; Supplementary Table S4 , available in the online version only), two domains were significantly different for the two age groups. Ambulation was mentioned less frequently in the older age group than in the younger age group, OR = 0.38, 95% CI (0.15, 0.94). By contrast, ADL dependency was mentioned more often in the older age group, OR = 3.51, 95% CI (1.20, 10.29). When comparing the domains considered burdensome for patients ( Fig. 4 ; Supplementary Table S4 , available in the online version only), no significant differences between the age groups were observed.

Regarding the ranking of the domains by the respondents for the different age groups ( Table 1 ), the overall responses were highly similar for younger and older patients.

### Effect of Country of Residence

Comparing the three largest respondent groups (Turkish, Italian, and Indian; Supplementary Tables S2, S3, S5, and S7 , available in the online version only), the differences found were limited. The burden of motor problems and decline or loss of ambulation was mentioned significantly less often in the Italian group compared with the Turkish group (OR: 0.15, 95% CI: 0.03–0.67, *p* = 0.01 and OR: 0.06, 95% CI: 0.01–0.36, *p* = 0.001, respectively). Cognitive symptoms were reported significantly more often in the Indian group than in the Turkish group (OR: 4.36, 95% CI: 1.42–13.34, *p* = 0.01; Supplementary Table S2 , available in the online version only). Regarding the burden to patients, ADL dependency was reported significantly more often in the Italian group than in the Turkish group (OR: 5.14, 95% CI: 1.29–20.52, *p* = 0.02; Supplementary Table S3 , available in the online version only). No further statistically significant differences were found for other symptom domains between populations, although small subgroup sizes and low event frequencies limited the statistical power for some comparisons.

Regarding the ranking of symptoms, motor function was identified as the most important domain in the Turkish, Italian, and Indian populations, followed by cognitive function and epilepsy control. Pain was most frequently identified as the least important domain in all three ethnic groups. In the Turkish and Italian populations, it was followed by sleep and then bladder and bowel control, whereas in the Indian population, the order was reversed.

### Hopes and Expectations

Supplementary Table S7 and Supplementary Figs. 1 and 2 (available in the online version only) summarizes the domains concerning respondents' hopes and expectations regarding therapy. The most frequently cited domain was improvement in motor function. Second in frequency were statements concerning the treatment and anticipated outcomes. Examples of these statements were halting the disease progression, painless therapeutic process, short treatment cycles, and avoiding treatment-related harm.

### Other

The final open question concerned other considerations for evaluating the efficacy of MLC treatment beyond those previously mentioned. Most respondents considered the inventory comprehensive.

## Discussion

Until now, there is no curative treatment for MLC. The majority of the patients have recessive variants in the gene MLC1. Recent studies show promising results of gene therapy in MLC1 knockout mice. 12 15 Additionally, G-protein coupled receptor (GPCR) subtype 5B (GPRC5B) has been shown as an interactor of MLC1, and variants in the gene GPRC5B may also cause MLC, linking this GPCR to MLC. 8 16 GPCRs are well-known drug targets, and targeting GPRC5B signaling is being investigated as a strategy for the treatment of MLC. 8 As advances in gene- and drug-based therapies for MLC begin to emerge, there is a growing need to establish outcome measures for future therapy trials. To date, clinical trial outcome measures have largely been determined by experts, with limited involvement from patients and their caregivers. Integrating the latter perspectives is crucial to ensure that therapeutic development reflects the priorities and lived experiences of those directly impacted by the disease. This study addresses this need by identifying the clinical outcomes and symptom domains that patients with MLC and their caregivers consider most important.

MLC places a substantial burden on patients and their families; the extent of this burden varies by disease subtype. A natural history reported disease signs and development in 242 MLC patients, of which 204 had classic and 38 had remitting MLC. 10 In patients with classic MLC, initial development, especially ambulation, is often normal or only mildly delayed. As the disease progresses, difficulties with ambulation, cognition, behavior, and seizure management emerge and become more prominent and therefore more burdensome. In patients with remitting MLC, ambulation is often delayed, but is typically acquired later. Epilepsy is less common and affects only 10% of patients. Cognitive impairment and autism are, however, common and may be severe. 10 These findings illustrate that the MLC phenotype impacts both the clinical profile and disease burden anticipated and experienced by patients and their families. Our study did not request genetic details, as many participants may not know this information, and it may be considered private. Consequently, our study could not distinguish between phenotypes. Given that by far most individuals with MLC have the classic phenotype, it is likely that our sample mainly represents this group.

The frequency of functional domains reported as most burdensome ( Figs. 1 to 4 ) and the ranking of the domains ( Table 1 ) reflect the clinical symptomatology of MLC. Motor disability is an early and consistent feature of MLC, affecting ambulation more than hand function. In line with this, motor function was consistently emphasized as the most burdensome domain across the entire cohort, all ethnic subgroups, and both age groups. Within this domain, decline or loss of ambulation was most frequently reported as particularly burdensome for both patients and caregivers. When ranking domains of burden, motor function was prioritized, and also respondents' hopes and expectations primarily regarded improvements in motor functioning. The ranking ( Table 1 ) of cognition and epilepsy as second and third also mirrors this pattern. Cognitive slowing and decline are consistent features of MLC, but manifest later in the disease course. Epilepsy is common, but typically not difficult to control with medication and therefore likely perceived as less burdensome. Pain ranked lowest ( Table 1 ), followed by sleep and bladder and bowel control, which is consistent with these not being common features of the disease. These findings were unexpected; we had anticipated that communication, ADL, and behavioral issues would be considered most critical, as decline and loss of ambulation can be amended with aids, like a walker or wheelchair, and are not critical for independent functioning.

To better understand how disease stage may affect perceived burden, we examined differences between younger (<15 years) and older (≥15 years) patients; the older patients generally had more severe motor impairment than the younger. 10 In our sample, the ages of the patients were distributed more or less evenly across the range. In both age groups, motor function, especially ambulation, was consistently mentioned as most burdensome. ADL dependency was, however, reported relatively more frequently as burdensome for caregivers of older patients than for those of younger patients. Limitations in ADL become more pronounced with increasing age of patients, making these domains more burdensome for caregivers to manage.

Perceptions of which symptoms are most burdensome and important to treat may also be influenced by cultural background and societal norms. The differences found were, however, limited, and respondents from different regions rated decline or loss of ambulation as the most important issue. Compared with respondents from Türkiye, caregivers of patients in Italy rated ADL dependency higher, while cognitive symptoms were mentioned more often in India. These differences should be interpreted with caution, given the small sample sizes. The absence of major differences in the responses suggests that translation and cultural factors are unlikely to have had a major impact on the results.

In the design of future clinical trials, selecting outcome measures that are both clinically meaningful and relevant to patients is of great importance. Ambulation represents a critical outcome measure, as decline or a loss of ambulation marks an important milestone in disease progression for MLC patients. Caregivers likewise identified ambulation as the most relevant domain, emphasizing its importance as an outcome measure that reflects both clinical and patient-centered priorities.
