# Supplementary material for: Patient and Family Perspectives on Clinical Outcomes in Therapy Trials in Megalencephalic Leukoencephalopathy with Subcortical Cysts: An Open Inventory
Source: Neuropediatrics. 2026 Apr 23;57(3):186–92. doi: 10.1055/a-2851-9956 (PMC13233118; doi:10.1055/a-2851-9956)
Supplement: Supplementary file 1 — Supplementary Material [file 10-1055-a-2851-9956-s1220254238oa.pdf]

Supplementary file

<u>Survey</u>

Title: What Signs or Symptoms Should Be Prioritized in the Treatment of MLC?

General information

- Are you the father, the mother, another legal guardian, or a patient?
  ☐ Father
  ☐ Mother
  ☐ Legal guardian
  ☐ Patient
- What is your country of residence?
- How old is your child with MLC? If you are a patient, fill in your own age.
  Years:
  Months:

Introduction

If treatment for MLC were developed, we would not expect that the disease would be completely cured, but rather that its progression would slow down, stop, or that some symptoms would partially improve. It is important that future treatments focus on the symptoms that matter most to patients and caregivers living with MLC. Therefore, we are asking you as patients and caregivers which symptoms of the disease are most important to you.

If you don't know the answer to a question or if you find it difficult to answer, you can write 'I don't know' and then move on to the next question.

Question 1

- *Could you describe which symptoms related to MLC are most burdensome in your daily life?*
- *And which are most burdensome in the daily life of the patient?*
- *Are those the items that should be considered to measure therapy effects?*
- *Which would you prioritize?*

Question 2

Below is a list of various signs and symptoms of MLC that could be used to measure the effects of treatment. Please rank these in order of importance, with 1 being the most important and 9 the least important. To register the answer, you must move at least one box. If the current order is to your liking, you can move a box back and forth.

- Motor functioning (e.g., walking unaided, walking with support, using a tablet, eating, drinking and dressing without help)
- Cognitive functioning (e.g., understanding situations, following a conversation)
- Behavior (e.g., restlessness, aggression, confusion, inactivity)
- Mood (e.g., depression, anger, happiness)
- Communication (e.g., making clear with speech or gestures what the problem is)
- Bladder and bowel control
- Control of epilepsy
- Sleep
- Pain

Please explain for each item what aspects you find important.

Question 3

What are your hopes and expectations regarding therapy?

Feel free to add anything that is on your mind.

Question 4

Are there any other aspects, not mentioned above, that should be considered when assessing the efficacy of MLC treatment?

Feel free to include as many items as you like.

Tables

Supplementary Table 1. Country of residence of respondents	
**Country of residence**	**Number (%)**
Argentina	1 (1)
Australia	1 (1)
Canada	3 (3.5)
France	3 (3.5)
Germany	2 (2.5)
India	16 (19)
Iran	1 (1)
Italy	17 (20)
Japan	4 (5)
Latvia	1 (1)
Nepal	1 (1)
Portugal	1 (1)
Spain	1 (1)
The Netherlands	4 (5)
Türkiye	23 (27)
United Kingdom	1 (1)
United States of America	5 (6)

Supplementary Table 2. Symptoms rated as burdensome related to MLC in daily life for caregivers in different populations

	Whole population (n=80)	Turkish population (n=20)	Italian population (n=16)		Indian population (n=16)	
**Domain**	**For caregivers Number (%)**	**For caregivers Number (%)**	**For caregivers Number (%)**	**Odds Ratio (95% CI)**	**For caregivers Number (%)**	**Odds Ratio (95% CI)**
Motor function	59 (74)	16 (80)*	6 (38)*	0.15 (0.03-0.67)	14 (88)	1.32 (0.53-3.32)
Decline or loss of ambulation	46 (58)	14 (70)*	2 (13)*	0.06 (0.01-0.36)	11 (69)	0.97 (0.48-1.98)
Unspecified motor problems	18 (23)	3 (15)	3 (19)	1.31 (0.23-7.57)	3 (19)	1.14 (0.48-2.75)
Manual dysfunction	5 (6)	1 (5)	0 (0)	-	0 (0)	-
Slow motor development	3 (4)	1 (5)	0 (0)	-	1 (6)	1.13 (0.27-4.69)
Communication	22 (28)	6 (30)	7 (44)	1.81 (0.46-7.18)	3 (19)	0.73 (0.33-1.62)
ADL dependency	19 (24)	5 (25)	8 (50)	3.00 (0.73-12.27)	2 (13)	0.66 (0.28-1.67)
Epilepsy	16 (20)	5 (25)	2 (13)	0.43 (0.07-2.58)	0 (0)	-
Cognition	15 (19)	1 (5)*	1 (6)	1.27 (0.07-21.97)	8 (50)*	4.36 (1.42-13.34)
Behavioral and psychiatric problems	13 (16)	2 (10)	2 (13)	1.29 (0.16-10.30)	4 (25)	1.73 (0.69-4.36)
Other symptoms	12 (15)	3 (15)	3 (19)	1.31 (0.23-7.57)	2 (13)	0.90 (0.34-2.35)
I don't know	1 (1)	1 (5)	0 (0)	-	0 (0)	-
No problems	0 (0)	0 (0)	0 (0)	-	3 (19)	-

Binary logistic regression was performed for the Turkish, Italian, and Indian cohorts; Türkiye served as the reference group (ORs and 95% CIs for Italy vs. Türkiye and India vs. Türkiye. *, Motor function: *OR* 0.15, 95% CI [0.03–0.68], $p = 0.01$; Ambulation: *OR* 0.06, 95% CI [0.01–0.36], $p = 0.001$; Cognition: *OR* 4.36, 95% CI [1.42–13.34], $p = 0.01$. This table is represented by Figure 2.

Supplementary Table 3. Symptoms rated as burdensome related to MLC in daily life for patients in different populations						
	**Whole population (n=85)**	**Turkish population (n=23)**	**Italian population (n=17)**		**Indian population (n=16)**	
**Domain**	**For patients Number (%)**	**For patients Number (%)**	**For patients Number (%)**	**Odds Ratio (95% CI)**	**For patients Number (%)**	**Odds Ratio (95% CI)**
Motor function	49 (58)	14 (61)	9 (53)	0.72 (0.20-2.57)	11 (69)	1.19 (0.60-2.33)
Decline or loss of ambulation	39 (46)	13 (57)	5 (29)	0.32 (0.09-1.21)	10 (63)	1.13 (0.59-2.18)
Unspecified motor problems	21 (25)	6 (26)	4 (24)	0.87 (0.20-3.74)	2 (13)	0.64 (0.27-1.53)
Manual dysfunction	10 (12)	2 (9)	2 (12)	1.40 (0.18-11.09)	0 (0)	-
Slow motor development	0 (0)	0 (0)	0 (0)	-	0 (0)	-
Communication	23 (27)	7 (30)	5 (29)	0.95 (0.24-3.75)	5 (31)	1.02 (0.51-2.03)
ADL dependency	19 (22)	5 (22)*	10 (59)*	5.14 (1.29-20.52)	2 (13)	0.72 (0.29-1.75)
Epilepsy	9 (11)	2 (9)	3 (18)	2.25 (0.33-15.24)	0 (0)	-
Cognition	7 (8)	0 (0)	1 (6)	-	3 (19)	-
Behavioral and psychiatric problems	10 (12)	4 (17)	0 (0)	0.00 (-)	2 (13)	0.82 (0.33-2.06)
Other symptoms	7 (8)	3 (13)	2 (12)	0.89 (0.13-6.01)	0 (0)	0.00 (-)
I don’t know	4 (5)	3 (13)	0 (0)	0.0 (-)	0 (0)	0.00 (-)
No problems	3 (4)	0 (0)	0 (0)	-	0 (0)	-

Binary logistic regression was performed for the Turkish, Italian, and Indian cohorts; Türkiye served as the reference group (ORs and 95% CIs for Italy vs. Türkiye and India vs. Türkiye. *, ADL dependency: *OR* 5.14, 95% CI [1.29–20.52], $p = 0.02$. This table is represented by Figure 3.

Supplementary Table 4. Symptoms rated as burdensome related to MLC in daily life in different age groups

	Population <15 years (n=48)	Population ≥ 15 years (n=32)		Population <15 years (n=49)	Population ≥ 15 years (n=36)	
**Domain**	**For caregivers Number (%)**	**For caregivers Number (%)**	**Odds Ratio (95% CI)**	**For patients Number (%)**	**For patients Number (%)**	**Odds Ratio (95% CI)**
Motor function	37 (77)	22 (69)	0.65 (0.24-1.78)	28 (57)	21 (58)	1.05 (0.44-2.51)
Decline or loss of ambulation	31 (65)*	13 (41)*	0.38 (0.15-0.94)	24 (49)	15 (42)	0.74 (0.31-1.77)
Unspecified motor problems	9 (19)	9 (28)	1.70 (0.59-4.88)	12 (24)	9 (25)	1.03 (0.38-2.78)
Manual dysfunction	1 (2)	4 (13)	6.71 (0.71-63.11)	5 (10)	5 (14)	1.42 (0.38-5.32)
Slow motor development	3 (6)	0 (0)	-	0 (0)	0 (0)	-
Communication	14 (29)	8 (25)	0.81 (0.29-2.23)	16 (33)	7 (19)	0.50 (0.18-1.38)
ADL dependency	7 (15)*	12 (38)*	3.51 (1.20-10.29)	8 (16)	11 (31)	2.26 (0.80-6.4)
Epilepsy	11 (23)	5 (16)	0.62 (0.19-2.00)	4 (8)	5 (14)	1.82 (0.45-7.30)
Cognition	9 (19)	6 (19)	1.00 (0.32-3.15)	3 (6)	4 (11)	1.92 (0.40-9.15)
Behavioral and psychiatric problems	8 (17)	5 (16)	0.93 (0.27-3.13)	5 (10)	5 (14)	1.42 (0.38-5.32)
Other symptoms	8 (17)	4 (13)	0.71 (0.20-2.6)	2 (4)	5 (14)	3.80 (0.69-20.78)
I don't know	1 (2)	0 (0)	-	4 (8)	0 (0)	-
No problems	0 (0)	0 (0)	-	1 (2)	2 (6)	2.82 (0.25-32.41)

Responses analyzed by age (<15 vs. ≥15 years) for caregivers and patient related questions. *, Ambulation: *OR* = 0.38, 95% CI [0.15, 0.94]; ADL dependency: *OR* = 3.51, 95% CI [1.20, 10.29]. This table is represented by Figure 4.

Supplementary Table 5. Ranking of the most and least important domains in different populations					
		**Whole population (n=85)**	**Turkish population (n=23)**	**Italian population (n=17)**	**Indian population (n=16)**
**Rank**	**Domain**	**Number (%)**	**Number (%)**	**Number (%)**	**Number (%)**
1	Motor function	59 (69)	20 (87)	11 (65)	10 (63)
2	Cognitive function	13 (15)	1 (4)	2 (12)	4 (25)
3	Control of epilepsy	7 (8)	1 (4)	2 (12)	1 (6.3)
…					
7	Bladder and bowel control	12 (14)	3 (13)	-	3 (19)
8	Sleep	15 (18)	7 (30)	4 (24)	1 (6)
9	Pain	38 (45)	11 (48)	5 (29)	10 (63)

The three most important and three least important domains are shown; "…" indicates the ranks in between. Domains include motor functioning, cognitive functioning, control of epilepsy, sleep, pain, and bladder/bowel control. For the domains mood, behavior, and communication the order differs per country and the numbers are close to each other and therefore not shown.

Thieme

Supplementary Table 6. Ranking of the most and least important domains in caregivers vs. patients					
**Caregivers (n=80)**			**Patients (n=5)**		
**Rank**	**Domain**	**Number (%)**	**Rank**	**Domain**	**Number (%)**
1	Motor function	54 (68)	1	Motor function	5 (100)
2	Cognitive function	13 (16)	…		
3	Control of epilepsy	11 (14)	…		
…			…		
7	Bladder and bowel control	12 (15)	…		
8	Sleep	14 (18)	…		
9	Pain	37 (46)	9	Control of epilepsy	3 (60)

The three most important and three least important domains are shown; "…" indicates the ranks in between. For the domains not shown the numbers are so low that the ranking is unreliable. For caregivers, these are mood, behavior, and communication. For the cognitive function, control of epilepsy, bladder and bowel control, sleep, and pain the numbers are very low and therefore not shown.

Supplementary Table 7. Hopes and expectations regarding therapy and treatment						
	**Whole population (n=85)**	**Turkish population (n=23)**	**Italian population (n=17)**	**Indian population (n=16)**	**Population <15 years (n=49)**	**Population ≥ 15 years (n=36)**
**Domain**	**Number (%)**	**Number (%)**	**Number (%)**	**Number (%)**	**Number (%)**	**Number (%)**
**Motor function**	**24 (28)**	**8 (35)**	**4 (24)**	**6 (38)**	**15 (31)**	**9 (25)**
Decline or loss of ambulation	23 (27)	6 (26)	4 (24)	6 (38)	15 (31)	5 (14)
Unspecified motor problems	10 (12)	3 (13)	2 (12)	2 (13)	5 (10)	8 (22)
Physical therapy and motor rehabilitation	1 (1)	0 (0)	1 (6)	0 (0)	0 (0)	1 (3)
Communication	14 (17)	1 (4)	4 (24)	4 (25)	7 (14)	7 (19)
ADL dependency	12 (14)	6 (26)	0 (0)	3 (19)	7 (14)	5 (14)
Other symptoms	9 (11)	3 (13)	2 (12)	0 (0)	6 (12)	3 (8)
Epilepsy	8 (9)	1 (4)	3 (18)	2 (13)	5 (10)	3 (8)
Cognition	6 (7)	0 (0)	0 (0)	3 (19)	5 (10)	1 (3)
Behavioral and psychiatric problems	5 (6)	3 (13)	0 (0)	1 (6)	3 (6)	2 (6)
Quality of Life	4 (5)	0 (0)	1 (6)	0 (0)	3 (6)	1 (3)
**Treatment design/effect**	**26 (31)**	**3 (13)**	**7 (41)**	**5 (31)**	**17 (35)**	**9 (25)**
I don't know	5 (6)	3 (13)	0 (0)	1 (6)	2 (4)	3 (8)
No problems	1 (1)	0 (0)	0 (0)	0 (0)	0 (0)	1 (3)
Miscellaneous	11 (12)	4 (17)	1 (6)	1 (6)	8 (16)	2 (6)
No other items	2 (2)	1 (4)	0 (0)	0 (0)	0 (0)	2 (6)

This table is represented by Supplementary Figures 1 and 2.

Figures

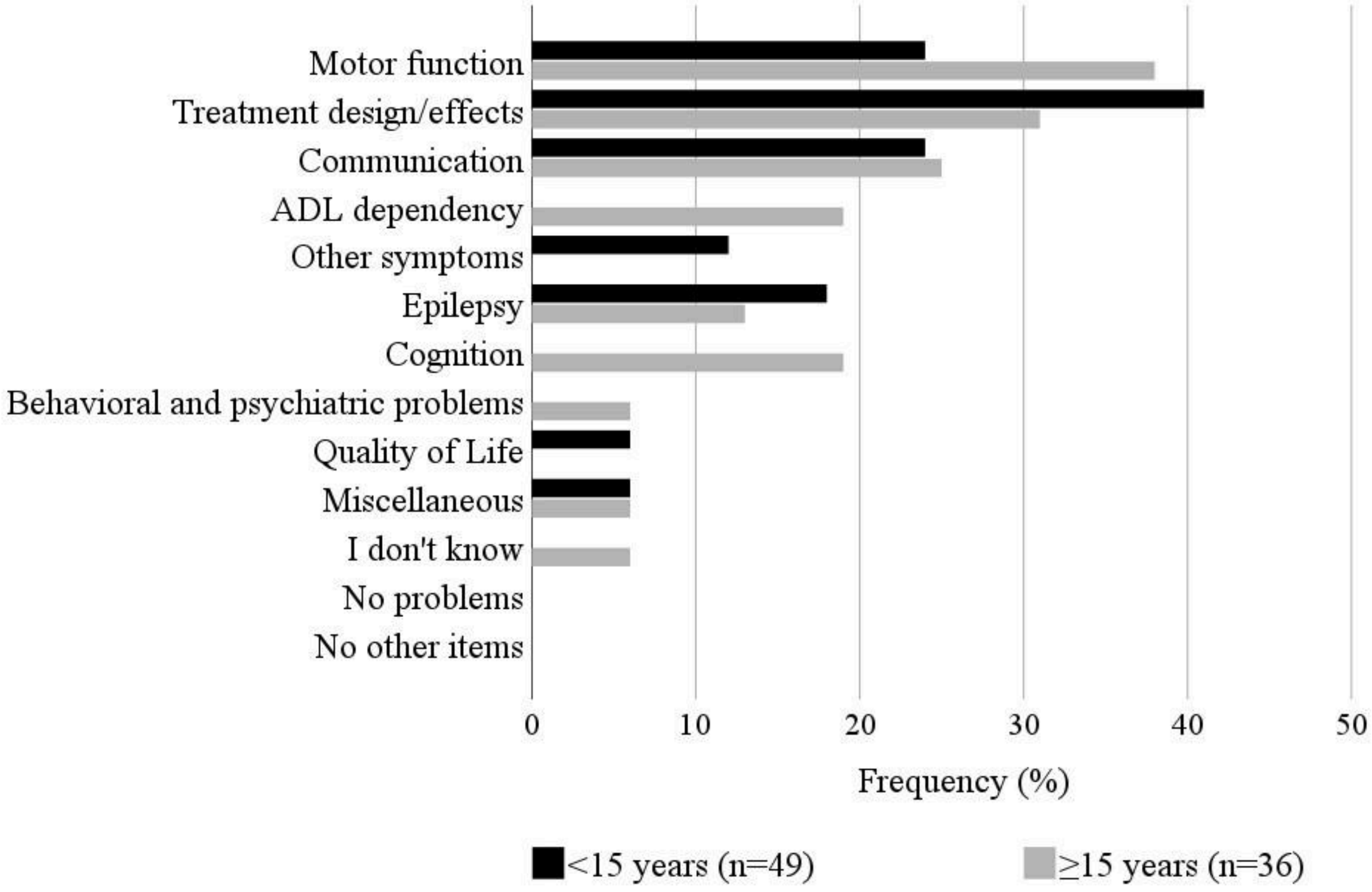

Supplementary Figure 1. Hopes and expectations regarding therapy and treatment across different populations. The frequency of reports per domain is shown on the x-axis, domains are displayed on the y-axis. This figure is represented by Supplementary Table 7.

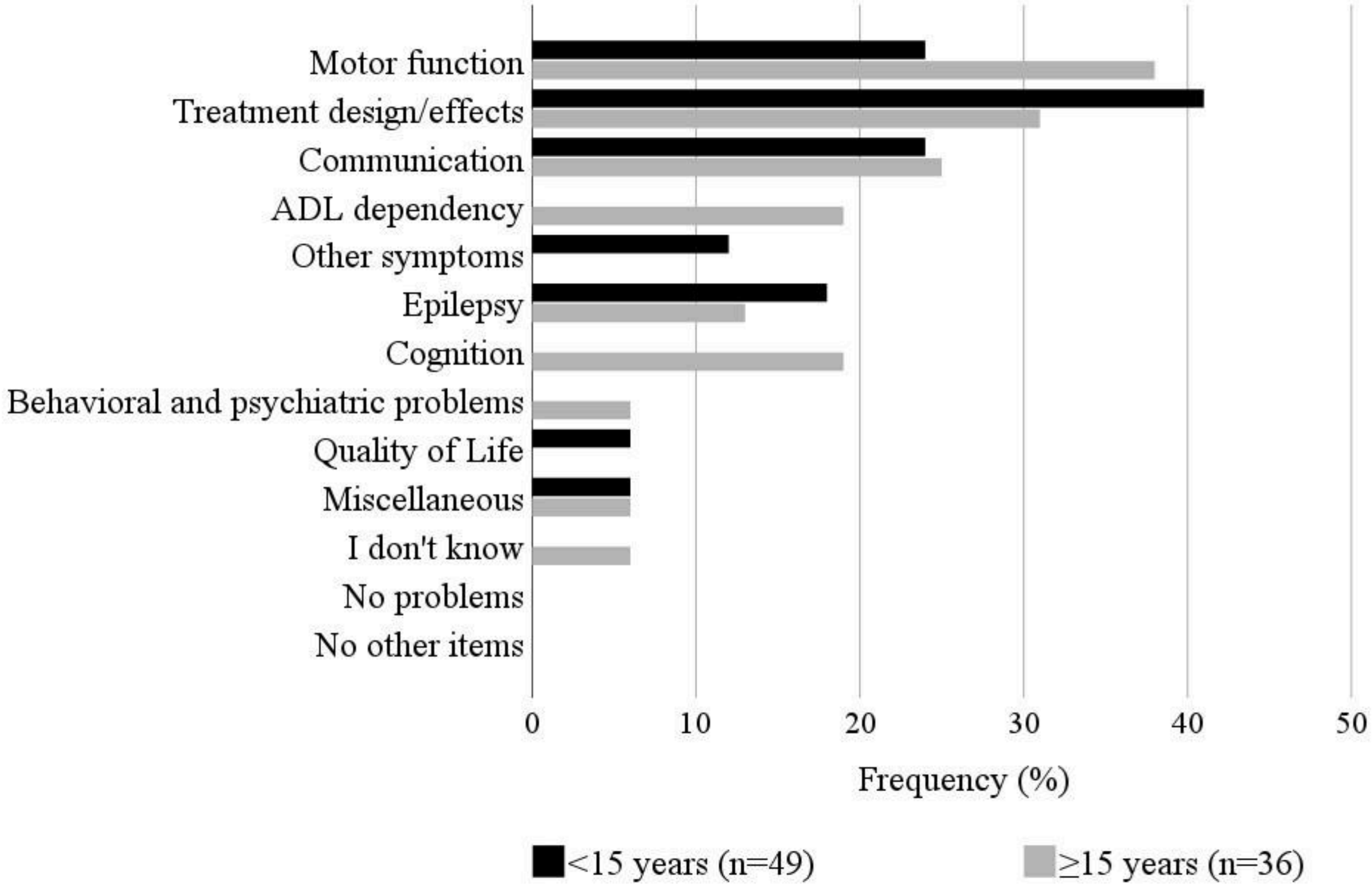

Supplementary Figure 2. Hopes and expectations regarding therapy and treatment across different age groups. The frequency of reports per domain is shown on the x-axis, with domains displayed on the y-axis. This figure is represented by Supplementary Table 7.
